# Biology, classification, and entomopathogen-based management and their mode of action on *Tuta absoluta* (Meyrick) in Asia

**DOI:** 10.3389/fmicb.2024.1429690

**Published:** 2024-08-07

**Authors:** Perumal Vivekanandhan, Kannan Swathy, Pittarate Sarayut, Krutmuang Patcharin

**Affiliations:** ^1^Research Administration Section, Chiang Mai University, Chiang Mai, Thailand; ^2^Department of Entomology and Plant Pathology, Faculty of Agriculture, Chiang Mai University, Chiang Mai, Thailand

**Keywords:** invasive insect pest, major pest of tomato, management, south American tomato leaf miner, *Tuta absoluta*

## Abstract

*Tuta absoluta*, known as the South American tomato leaf miner, significantly impacts tomato plants (*Solanum lycopersicum*) economically on a global scale. This pest, belonging to the Gelechiidae family, is native to South America and was first identified in Peru in 1917. Since its discovery, *T. absoluta* has rapidly spread to Europe, Africa, and Asia, severely threatening tomato production in these regions. The widespread application of chemical pesticides against this pest has resulted in significant environmental harm, including contamination of soil and water, and has had negative effects on non-target species such as beneficial insects, birds, and aquatic life. Although substantial research has been conducted, biological control methods for *T. absoluta* remain insufficient, necessitating further study. This review covers the Biology, Classification, and Entomopathogen-Based Management of *T. absoluta* (Meyrick) in Asia. It provides essential insights into the pest’s life cycle, ecological impacts, and the potential of entomopathogens as biocontrol agents. The detailed information presented aims to facilitate the development of sustainable pest control strategies, minimizing environmental impact and promoting the use of entomopathogens as viable alternatives to chemical pesticides in controlling *T. absoluta* insect pest.

## 1 Introduction

Invasive insect pests pose significant threats to global agricultural food production, exacerbated by factors such as climate change and the international trade of agricultural commodities (Skendžić et al., 2021). *Tuta absoluta* (Meyrick, 1917) (Lepidoptera: Gelechiidae) exemplifies this challenge as a devastating pest of tomato (*S. lycopersicum*) and other solanaceous crops. The economic impact of *T. absoluta* is profound, with substantial global expenditures incurred for its control and the mitigation of crop losses (Vivekanandhan et al., 2024a,b,c). For instance, Turkey spends approximately $183.7 million USD annually on *T. absoluta* control (Oztemiz, 2014), while Nepal reported crop losses totaling $19.7 million in the initial year of the invasion (Bajracharya et al., 2016). These losses have significant socio-economic repercussions, including a substantial 32% surge in tomato prices (Vivekanandhan et al., 2024a,b,c).

The global spread of *T. absoluta* has been rapid and extensive, impacting tomato production across continents (Fiaboe et al., 2021; Ndiaye et al., 2021; Vivekanandhan et al., 2024a,b,c). Initially detected in Spain in 2006 (Campos et al., 2017), *T. absoluta* has since spread to Africa, Eurasia, and Western Africa following its introduction to Niger in 2012 (Biondi et al., 2018). In Asia, the pest was first identified in Turkey in 2009 (Kılıç, 2010) and subsequently reported in Taiwan (2020), Bangladesh, Nepal (2016), Myanmar (2017), and regions of China (2017–2018) (Ramasamy, 2020; Yule et al., 2021; Zhang et al., 2021). The movement of tomato seedlings and fruits through international trade routes has facilitated its dispersal in Asia (Guimapi et al., 2020). To effectively manage the spread of *T. absoluta* and mitigate its impact on non-infested regions in Asia such as Bhutan, and North Korea stringent quarantine measures and phytosanitary protocols are imperative. Understanding the pest’s biology, climatic preferences, and pathways of human-mediated dispersal are crucial for assessing invasion risks and developing sustainable management strategies (Banks et al., 2015).

Research focused on the biology, ecological impact, spread dynamics, and control tactics against *T. absoluta* in Asia is essential for mitigating the persistent threat posed by this invasive species. Non-infested countries must prioritize proactive measures to prevent the introduction of *T. absoluta* and safeguard their agricultural industries from potential disruptions and economic losses. By leveraging scientific knowledge and fostering international cooperation, we can effectively reduce the risk of *T. absoluta* invasion while promoting sustainable agricultural practices globally.

## 2 Scientific classifications

*Tuta absoluta*, commonly known as the tomato leafminer, is classified within the domain Eukaryota and kingdom Animalia. It belongs to the phylum Arthropoda and class Insecta. This species is part of the order Lepidoptera and family Gelechiidae (Table 1). Within this family, it is placed in the genus Tuta, with its species designation being *T. absoluta*. This moth is a significant agricultural pest, particularly affecting Solanaceae crops.

**Table 1:** The scientific classification of *T. absoluta*, commonly known as the tomato leafminer.

***Tuta absoluta* scientific classification**	
Domain	Eukaryote
Kingdom	Animalia
Phylum	Arthropoda
Class	Insecta
Order	Lepidoptera
Family	Gelechiidae
Genus	Tuta
Species	*T. absoluta*
Binomial name	*Tuta absoluta*

### 2.1 *Tuta absoluta* biology

**Figure 1:**
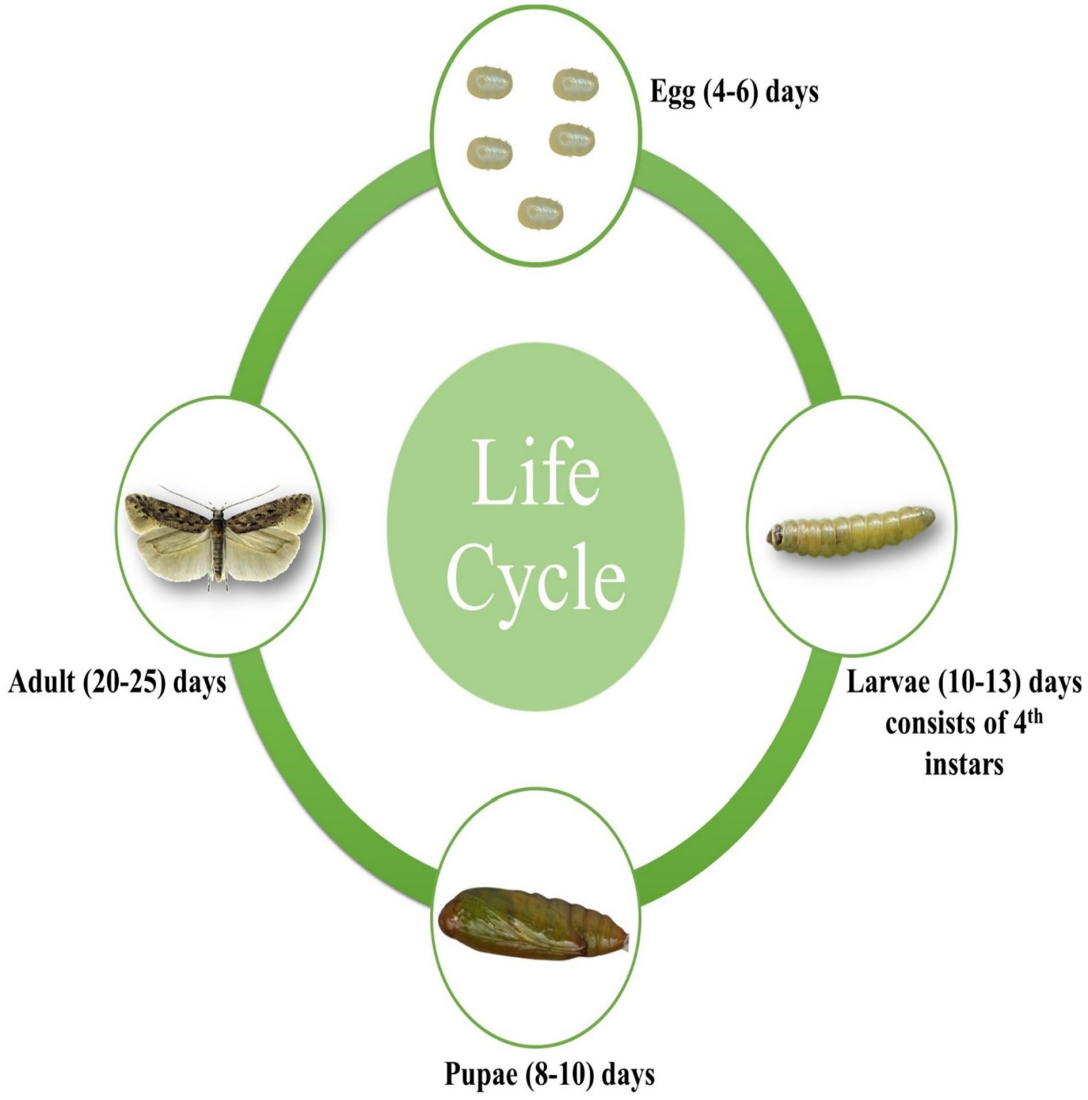
Life cycle of *T. absoluta* (Meyrick) (Lepidoptera; Gelechiidae).

*Tuta absoluta*, a holometabolous insect, has a complex life cycle encompassing four distinct stages: egg, larva, pupa, and adult (Figure 1). Each stage exhibits unique morphological and behavioral characteristics. Understanding these stages in detail is essential for developing effective and targeted pest management strategies, thereby mitigating the significant economic impact on tomato production.

#### 2.1.1 Egg stage

Adult female *T. absoluta* deposit yellow, elliptical eggs (0.33 × 0.22 mm) on the upper surfaces of their host plants, such as sepals, young leaves, or stems (Figure 1). Each female can lay approximately 260 eggs during her lifetime (Uchoa-Fernandes et al., 1995). Under favorable conditions, the eggs hatch within 4–6 days in tomato plants.

#### 2.1.2 Larval stage

The larvae of *T. absoluta* are highly destructive, causing significant damage to plant foliage by mining through the mesophyll layer of leaves and later penetrating auxiliary buds and fruits, resulting in yield losses (Cocco et al., 2015). The larval stage consists of four instars, with body lengths progressively increasing from 2.8 mm to 7.7 mm (Colmenárez et al., 2022). Larvae change color from white in the early instars to light green in later stages. Under favorable conditions, the larval stage lasts 10–13 days in tomato plants.

#### 2.1.3 Pupal stage

After completing their larval development, mature *T. absoluta* larvae typically drop to the soil to pupate, although pupation can also occur on plant leaves. The pupae are initially green but gradually turn dark brown (Figure 1). Mature pupae measure approximately 4.35 mm in length and 1.1 mm in width (Colmenárez et al., 2022). Under favorable conditions, the pupal stage lasts 8–10 days in tomato plants.

#### 2.1.4 Adult stage

According to Colmenárez et al. (2022), adult *T. absoluta* moths are approximately 6 mm long, with dark gray coloration and brown and off-white scales. Nocturnal by nature, they hide among leaves during the day (EPPO, 2005). The duration of each life stage varies with environmental conditions (Figure 1). Erdogan and Babaroglu (2014) reported that at 25°C and 65% relative humidity, the egg, larval, and pupal stages last about 4.1, 11.0, and 9.5 days, respectively, resulting in an egg-to-adult lifespan of approximately 30.2 days. Under favorable conditions, the adult stage lasts 20–25 days in tomato plants.

### 2.2 Life cycle and reproduction

The complete life cycle of *T. absoluta* typically spans between 29 to 38 days, with variability influenced significantly by environmental conditions such as temperature and humidity (EPPO, 2005). Adult males and females of *T. absoluta* have relatively short lifespans, with males surviving approximately 15.8 days and females about 18.2 days on average. The oviposition period lasts around 7.9 days, during which females can lay up to 141 eggs each (Erdogan and Babaroglu, 2014; Vivekanandhan et al., 2024a,b,c). However, EPPO (2005) suggests a higher fecundity rate, reporting that females may lay up to 260 eggs over their lifetime. The combination of high reproductive capacity and short generation time enables *T. absoluta* to undergo rapid population growth and inflict severe damage on tomato and other solanaceous crops. This pest’s ability to complete multiple generations in a single growing season further exacerbates its impact on agricultural productivity.

#### 2.2.1 Effect of hot climatic conditions on *Tuta absoluta* development

Temperature profoundly influences the growth, development, and behavior of *T. absoluta*, a significant insect pest impacting tomato and solanaceous crops. Studies have extensively examined how temperature affects various stages of its life cycle, revealing the species’ remarkable adaptability to thermal conditions (Van Damme et al., 2015). Cuthbertson et al. (2013) identified the optimal temperature range for *T. absoluta* development as 19–23°C, with egg hatching rates peaking at 13°C and adult emergence rates at 19°C. Temperatures below 10°C were found to result in developmental failure, highlighting the pest’s sensitivity to cold conditions. Conversely, Martins et al. (2016) reported an optimal temperature of 30°C for *T. absoluta* development, with lower and upper thresholds of 14°C and 34.6°C, respectively, indicating considerable variability in thermal preferences.

*Tuta absoluta’s* ability to undergo multiple generations per year without diapause further underscores its adaptability (EPPO, 2005; Biondi et al., 2018). Overwintering studies in Western Europe, particularly in greenhouses, reveal its persistence during colder months. Research on cold resistance shows larvae, pupae, and adults can withstand temperatures as low as −18.2°C, −16.7°C, and − 17.8°C, respectively (Van Damme et al., 2015). Moreover, LT_50_ values at 0°C indicate varying cold tolerance among life stages, with adults exhibiting higher resistance compared to larvae and pupae.

Unlike many insects, *T. absoluta* does not enter reproductive diapause in response to seasonal changes in temperature and day length, enhancing its ability to thrive in temperate climates (Van Damme et al., 2015). These adaptive traits contribute to its widespread distribution and ability to inflict substantial economic losses year-round. Understanding the thermal biology and adaptive mechanisms of *T. absoluta* is crucial for devising effective integrated pest management strategies tailored to mitigate its impact on tomatoes and other host crops across diverse environmental conditions.

#### 2.2.2 Effect of humidity on *Tuta absoluta* development

Humidity plays a crucial role in the development and population dynamics of *T. absoluta*, the tomato leafminer (Kachave et al., 2020; Vivekanandhan et al., 2024a,b,c). This pest thrives in environments with moderate to high humidity levels, which are conducive to its reproductive success and overall lifecycle (Buragohain et al., 2021). High humidity enhances the survival and growth rates of *T. absoluta* eggs and larvae, facilitating faster development through its various life stages (Kachave et al., 2020; Vivekanandhan et al., 2024a,b,c). However, excessively high humidity levels can also favor the proliferation of fungal pathogens that affect *T. absoluta* populations. Conversely, low humidity conditions can impede egg hatching and larval development, thereby potentially reducing pest pressure on crops.

#### 2.2.3 Host plants of *Tuta absoluta*

*Tuta absoluta* is a polyphagous pest with a broad host range primarily within the Solanaceae family. It significantly impacts economically important crops such as tomato, potato, brinjal, sweet pepper, and tobacco (Mohamed et al., 2015; Abbes et al., 2016; Vivekanandhan et al., 2024a,b,c). Abbes et al. (2016) identified *Solanum nigrum* (European black nightshade) as particularly susceptible to *T. absoluta* infestations. Furthermore, this pest has been documented to harm plants from diverse families including Malvaceae, Amaranthaceae, Fabaceae, and Convolvulaceae, indicating its polyphagous behavior and adaptability to various agricultural and weed species (Bawin et al., 2016).

**Figure 2:**
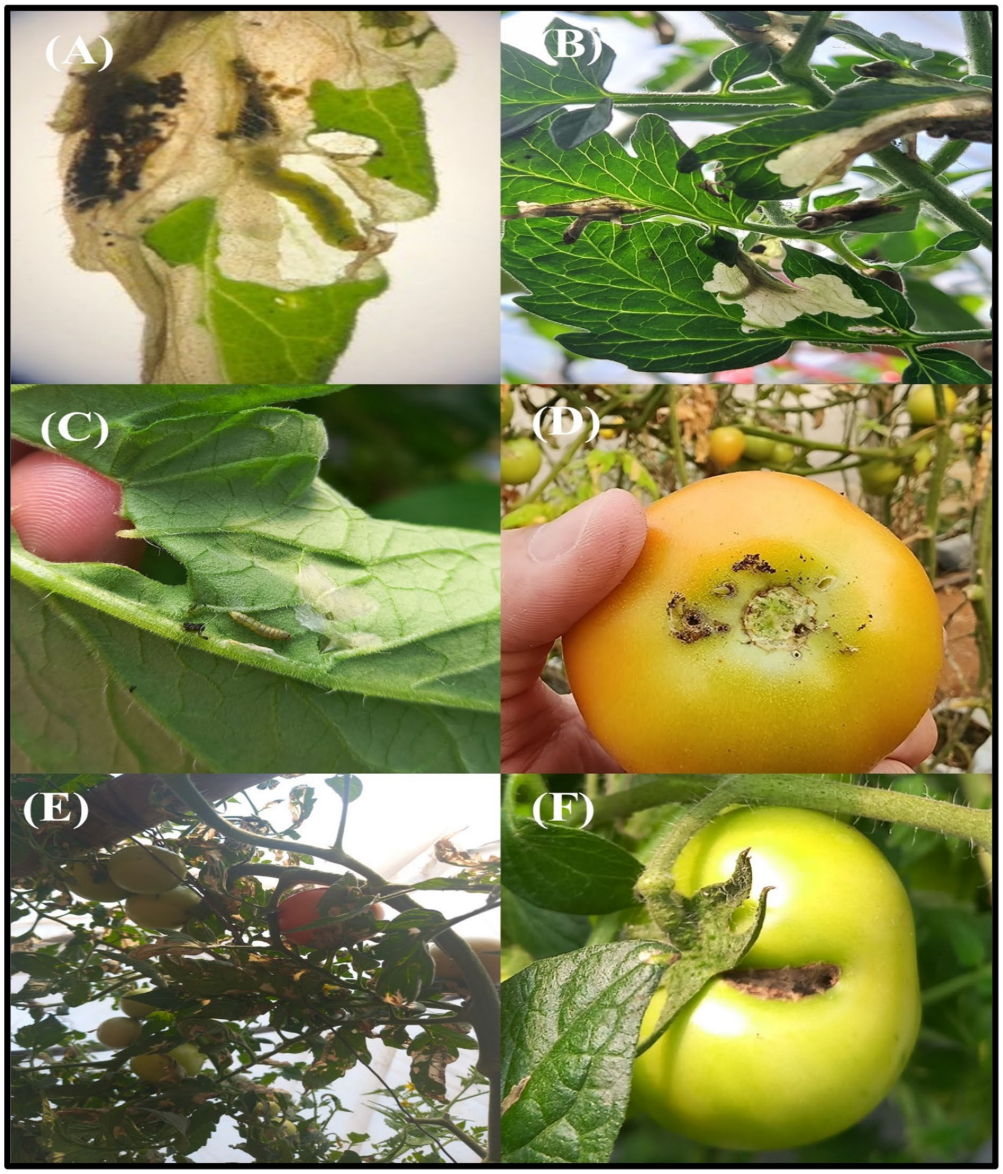
Symptoms of *T. absoluta* infection in tomato plants. *T. absoluta* damage in tomato plants and their parts **(A–F)**.

*Tuta absoluta* is recognized as a highly destructive pest that imposes significant economic losses in tomato farming (Figures 2A–F). In both greenhouse and open field environments, unchecked infestations of *T. absoluta* can result in yield reductions ranging from 80 to 100% (Figures 2A–F). The pest typically establishes colonies on tomato plants shortly after transplanting and reaches peak infestation levels during flowering and fruiting stages (Figures 2A–F). Diatte et al. (2018) documented the highest rates of *T. absoluta* infestation during the early fruiting stage, followed by early flowering, vegetative growth, and harvesting stages.

Research in Nepal by Bajracharya et al. (2018) highlighted varying degrees of damage caused by *T. absoluta* across different tomato varieties. The Karita variety suffered extensive damage ranging from 76 to 100%, while the Samjhana and Srijana varieties exhibited damage levels between 51 and 75%. This variability underscores the importance of understanding host susceptibility and emphasizes the need for selecting resistant or tolerant tomato cultivars as part of integrated pest management strategies. The infestation patterns and damage severity associated with *T. absoluta* underscore its impact on global tomato production.

## 3 Invasion in Asian countries

**Figure 3:**
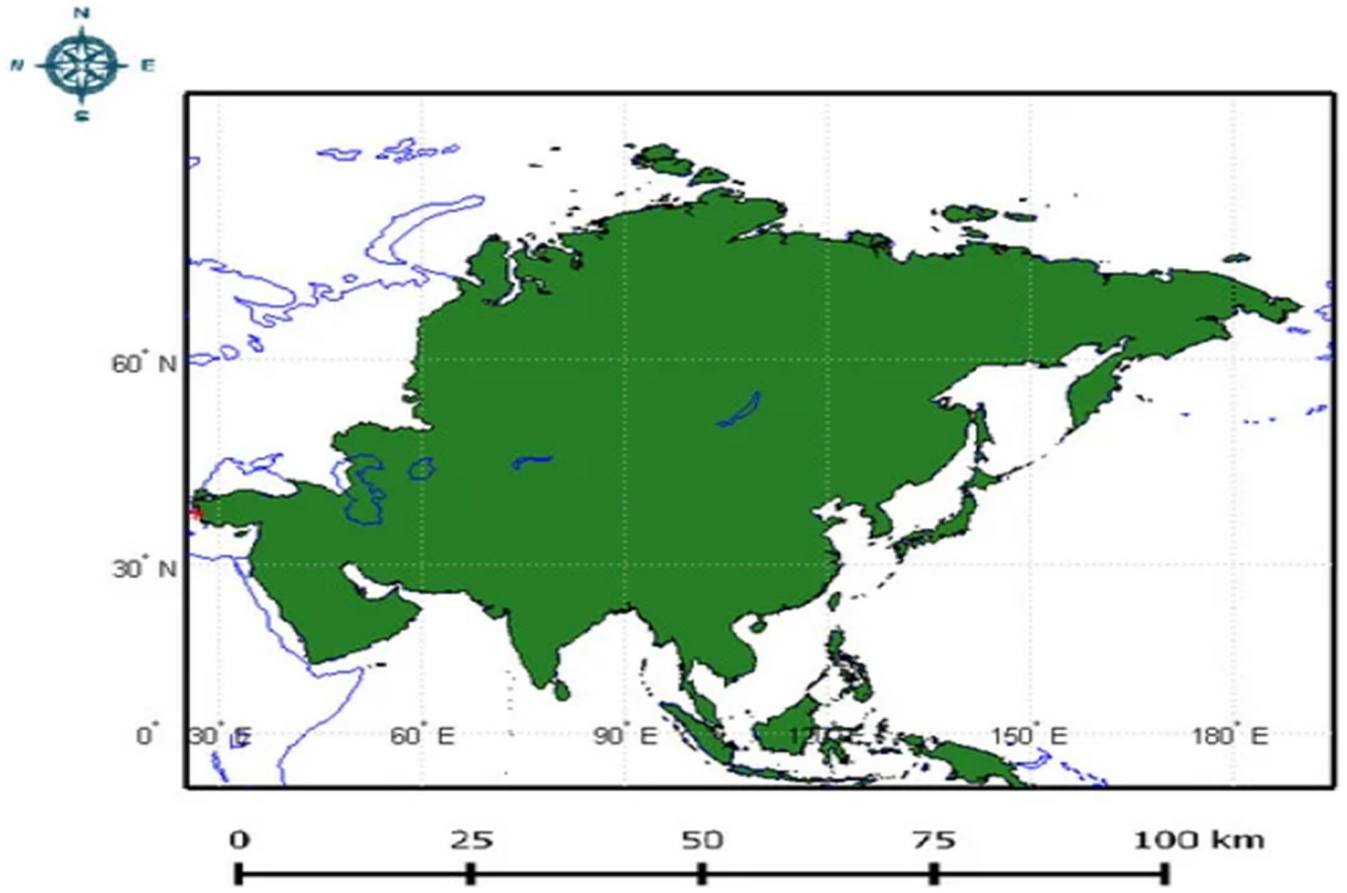
Depicts the Asian continent highlighted in green, with a red dot indicating the location in Turkey where *T. absoluta* was first discovered in 2009. This location marks the initial entry point of *T. absoluta* into Asia (Adapted from Guimapi et al., 2020).

The invasion of *T. absoluta* in Asian countries has profoundly affected agriculture and economies since its initial appearance. The pest was first detected in Turkey in 2009 and has subsequently spread across a wide swath of Asia, including Iran, Kazakhstan, Afghanistan, Lebanon, Bangladesh, Myanmar, Bahrain, Pakistan, Iraq, Turkmenistan, China, Kuwait, India, Nepal, Israel, Jordan, Kyrgyzstan, Qatar, Saudi Arabia, Syria, Tajikistan, United Arab Emirates, Uzbekistan, and Yemen (Guimapi et al., 2020; EPPO, 2023) (Figure 3).

In India, *T. absoluta* was first reported in 2014 in Maharashtra and has since spread to key tomato-growing regions like Karnataka, Tamil Nadu, Gujarat (Ballal et al., 2016), Andhra Pradesh, Telangana (Kumari et al., 2014), New Delhi (Shashank et al., 2016), Madhya Pradesh (Swathi et al., 2017), Punjab (Sidhu et al., 2017), Meghalaya (Sankarganesh et al., 2017), Himachal Pradesh (Sharma and Gavkare, 2017), and Uttarakhand (Singh and Panchbhaiya, 2018). The exact entry route into India remains uncertain, likely facilitated by unrestricted agricultural trade between states and prevailing wind patterns (Shashank et al., 2016). In May 2016, Bangladesh recorded its first instance of *T. absoluta* in tomato fields in Panchagarh district, swiftly spreading to neighboring districts (Hossain et al., 2016).

China documented infestations in the Ili Kizakg and Ili Xinjiang regions, causing significant damage to tomato, potato, and eggplant crops (Zhang et al., 2020). Taiwan faced invasion by *T. absoluta* in June 2020 (Ramasamy, 2020), while Myanmar reported varying infestation levels from 10 to 82% (Yule et al., 2021). Southeast Asian and Pacific nations like Indonesia, Korea, Japan, and Australia have not officially reported *T. absoluta* invasion but remain susceptible due to extensive trade in tomatoes and related crops with affected regions (McNitt et al., 2019; El-Shafie, 2020; Zhang et al., 2021).

## 4 *Tuta absoluta* management

Management strategies for *T. absoluta* utilizing entomopathogens offer a broad array of effective options, encompassing various biological agents such as entomopathogenic fungi (e.g., *Beauveria* spp., *Metarhizium* spp.), bacteria (e.g., *Bacillus thuringiensis*), viruses (e.g., nucleopolyhedroviruses), and nematodes (e.g., *Steinernema* spp.). These agents exhibit efficacy against multiple life stages of the *T. absoluta* insect pest, including eggs, larvae, pupae, and adults (see Table 2). Their application with entomopathogens based management programs provides sustainable alternatives to chemical pesticides, contributing to environmentally friendly and economically viable pest control strategies.

**Table 2:** Entomopathogens against tomato insect pest *T. absoluta.*

**S. no**	**Entomopathogens**	**Test concentration**	**Effective within**	**Host**	**Reference**
**Entomopathogenic fungi**					
1	*B. bassiana*	150 μg/mL	24 h	Larvae	Vivekanandhan et al. (2024a,b,c)
2	*V. lecanii*	1 × 10^3^ spore/ml	4 Days	Eggs	Abdel-Raheem (2020)
3	*B. bassiana*	1 × 10^3^ spore/ml	4 days	Eggs	Abdel-Raheem (2020)
4	*M. anisopliae*	0.5 × 10^9^ conidia/g 1 × 10^8^ conidia.ml^−1^	5 days	Adults pre-pupae	Akutse et al. (2020)
5	*B. bassiana*	10^8^ spores/ml	6 days	Larvae	Ndereyimana et al. (2019)
6	*B. bassiana*	10^8^ spores/ml	6 days	Larvae	Ndereyimana et al. (2019)
7	*M. anisopliae*	10^8^ spores/ml	5 days	Larvae	Ndereyimana et al. (2019)
8	*M. anisopliae*	5 × 10^8^	3–6 days	Pupae	Erasmus et al. (2021)
9	*M. anisopliae*	1.615 × 10^7^	3–6 days	Pupae	Erasmus et al. (2021)
10	*B. bassiana*	2.75 × 10^8^	3–6 days	Pupae	Erasmus et al. (2021)
11	*B. bassiana*	5.48 × 10^5^	3–6 days	Pupae	Erasmus et al. (2021)
12	*Aspergillus oryzae*	1.0 × 10^8^ conidia mL^−1^	2–3 days	Larvae, pupae and adult	Zekeya et al. (2019)
13	*Aspergillus oryzae*	1.0 × 10^8^ conidia mL^−1^		Larvae, pupae and adult	Zekeya et al. (2019)
14	*B. bassiana*	1 × 10^8^ conidia mL^−1^	6 days	Larvae	Silva et al. (2020)
15	*B. bassiana*	1 × 10^8^ conidia mL^−1^	7 days	Larvae	Silva et al. (2020)
16	*B. bassiana*	1 × 10^8^ conidia mL^−1^	8 days	Larvae	Silva et al. (2020)
17	*B. bassiana*	1 × 10^7^ spores /ml	6 days	Larvae	Hammad et al. (2021)
18	*B. bassiana*	1 × 10^7^ spores /ml	6 days	Larvae	Hammad et al. (2021)
19	*B. bassiana*	1 × 10^7^ spores /ml	7 days	Larvae	Hammad et al. (2021)
20	*P. lilacinum*	1 × 10^7^ spores /ml	6 days	Larvae	Hammad et al. (2021)
21	*B. bassiana*	2 × 10^8^ conidia/ml	3 days	Larvae	Karaca et al. (2022)
22	*I. fumosorosea*	2 × 10^8^ conidia/ml	7 days	Larvae	Karaca et al. (2022)
23	*P. lilacinum*	2 × 10^8^ conidia/ml	7 days	Larvae	Karaca et al. (2022)
24	Metarhizium species	2 × 10^8^ conidia/ml	7 days	Larvae	Karaca et al. (2022)
25	*M. anisopliae*	10^7^ conidia/ml	8 days	Larvae	Alikhani et al. (2019)
26	*B. bassiana*	2.5 × 10^7^ spores/ml	3 days	Larvae	El-Hindi (2016)
27	*Beauveria bassiana*	2.5 × 10^9^ conidia/ml^−1^	5 days	Larvae	Tadele and Emana (2017)
28	*M.* *anisopliae*	2.5 × 10^9^ conidia/ml^−1^	5 days	Larvae	Tadele and Emana (2017)
29	*B. bassiana*	10^10^ spores/ml	5 days	Eggs and larvae	Shalaby et al. (2013)
30	*M. anisopliae*	10^10^ spores/ml	5 days	Eggs and larvae	Shalaby et al. (2013)
31	*B. bassiana* (Sn182)	4 × 10^7^ spores/ml	24 h	Larvae	Mohamed Mahmoud et al. (2021)
32	*Clonostachys* species	4 × 10^7^ spores/ml	24 h	Larvae	Mohamed Mahmoud et al. (2021)
33	*M. anisopliae*	10^6^ conidia/mL	72 h	Eggs	Pires et al. (2009)
34	*M. anisopliae*	5.5 × 10^9^ conidia/mL	14 Days	Larvae	Bayram (2019)
35	*B. bassiana*	4× 10^9^ conidia/ml	7 Days	Larvae	Bayram (2019)
**Entomopathogenic bacteria**					
36	*B. thuringiensis*	10 × 2^8^ spores/ml	1–2 days	Larvae	Giustolin et al. (2001)
37	*B. thuringiensis*	2 g/L^−1^	4 weeks	Larvae	González-Cabrera et al. (2011)
38	*B. thuringiensis*	2 g/L^−1^	4 weeks	Larvae	González-Cabrera et al. (2011)
39	*B. thuringiensis*	2 g/L^−1^	4 weeks	Larvae	González-Cabrera et al. (2011)
40	*B. thuringiensis*	1.84× 10^6^ spores/ml	3 days 4 days 3 days 2 days 2 days	Neonate larvae First instar Second instar Third instar Forth instar	Giustolin et al. (2001)
41	*B.* *Thuringiensis*	10^10^ spores/ml	4 days	Larvae	Shalaby et al. (2013)
42	*B. thuringiensis*	2 × 10^9^ cfu/mL	3 days	Larvae	Eski et al. (2024)
43	*Staphylococcus petrasii*	1 × 10^9^ cfu/mL	3 days	Larvae	Eski et al. (2024)
44	*Citrobacter freundii*	1 × 10^9^ cfu/mL	3 days	Larvae	Eski et al. (2024)
45	*Chishuiella changwenlii*	1 × 10^9^ cfu/mL	3 days	Larvae	Eski et al. (2024)
46	*E. casseliflavus*	1 × 10^9^ cfu/mL	3 days	Larvae	Eski et al. (2024)
47	*P. tremae*	1 × 10^9^ cfu/mL	3 days	Larvae	Eski et al. (2024)
**Entomopathogenic nematodes**					
48	*H. bacteriophora*	50 IJs/50 μL	48 h	Larvae	El Aimani et al. (2021)
49	*H. bacteriophora*	50 IJs/50 μL	48 h	Larvae	El Aimani et al. (2021)
50	*H. bacteriophora*	50 IJs/50 μL	48 h	Larvae	El Aimani et al. (2021)
51	*S. feltiae*	200 IJs/ml^−1^	3 days	Larvae	Yüksel (2022)
52	*H. bacteriophora*	200 IJs/ml^−1^	2 days	Larvae	Yüksel (2022)
53	*H. bacteriophora*	200 IJs/ml^−1^	3 days	Larvae	Yüksel (2022)
54	*S. feltiae*	200 IJs/ml^−1^	3 days	Larvae	Yüksel (2022)
55	*S. feltiae*	200 IJs/ml^−1^	3 days	Larvae	Yüksel (2022)
56	*S. feltiae*	200 IJs/ml^−1^	3 days	Larvae	Yüksel (2022)
57	*S. feltiae*	200 IJs/ml^−1^	3 days	Larvae	Yüksel (2022)
58	*S. feltiae*	200 IJs/ml^−1^	2 days	Larvae	Yüksel (2022)
59	*S. feltiae*	200 IJs/ml^−1^	2 days	Larvae	Yüksel (2022)
60	*S. feltiae*	200 IJs/ml^−1^	2 days	Larvae	Yüksel (2022)
61	*S. feltiae*	200 IJs/ml^−1^	2 days	Larvae	Yüksel (2022)
62	*Steinernema feltiae*	50 IJs/50 μL	48 h	Larvae	El Aimani et al. (2021)
63	*S. feltiae*	50 IJs/50 μL	48 h	Larvae	El Aimani et al. (2021)
64	*S. carpocapsae*	50 IJs/cm^2^	15 days	Larvae	Gözel and Kasap (2015)
65	*H.* *bacteriophora*	50 IJs/cm^2^	9 Days	Larvae	Gözel and Kasap (2015)
66	*Steinernema affine*	50 IJs/cm^2^	15 days	Larvae	Gözel and Kasap (2015)
67	*Steinernema feltiae*	50 IJs/cm^2^	3 Days	Larvae	Gözel and Kasap (2015)
**Entomopathogenic virus**					
68	PhopGV	5.54 × 10^7^ OBs/ml^−1^	13 days	Larvae	Mascarin et al. (2010)
69	*Colombian granuloviruses*	1 × 10^9^ OBs/ml^−1^	14 days	Larvae	Gómez Valderrama et al. (2018)
70	*Colombian granuloviruses*	1 × 10^9^ OBs/ml^−1^	8.6 days	Larvae	Gómez Valderrama et al. (2018)

### 4.1 Entomopathogenic fungi and bacteria

Entomopathogenic fungi (EPF) are heterotrophic, eukaryotic filamentous microorganisms that reproduce conidia either sexually or asexually (Mora et al., 2017; Vivekanandhan et al., 2021, 2024a,b). The majority of EPF, including *Beauveria bassiana, Metarhizium anisopliae*, *Metarhizium acridum*, *Metarhizium brunneum*, *Isaria fumosorosea*, *Hirsutella thompsonii*, and *Lecanicillium lecanii*, are classified as Ascomycetes and highly virulent to a broad range of medical and agricultural insect pests (Dara, 2017; Vivekanandhan et al., 2022a,b, 2023, 2024a,b,c; Swathy et al., 2023, 2024; Krutmuang et al., 2024; Perumal et al., 2024a,b). The fungi are pathogenic to various insect genera, causing muscardine disease in a wide range of hosts with minimal environmental impact and insect resistance (El-Hindi, 2016; Krutmuang et al., 2023; Perumal et al., 2023a,b). Although the efficacy of these entomopathogenic fungi depends on environmental conditions, *B. bassiana* and *M. anisopliae* are the most extensively researched and commercialized fungal species (Vivekanandhan et al., 2020; Kannan et al., 2023; Vivekanandhan et al., 2024a,b,c). These EPF demonstrated high larval mortality against several agriculturally important insect pests.

Studies on the effectiveness of *B. bassiana* and *B. thuringiensis* against *T. absoluta* have demonstrated varying levels of vulnerability across larval stages. González-Cabrera et al. (2011) and Alsaedi et al. (2017) found that first instar larvae were the most susceptible to *B. thuringiensis*, aiding in keeping *T. absoluta* populations below economic thresholds. In contrast, research indicated that third instar larvae were particularly vulnerable to both *B. bassiana* and *B. thuringiensis*.

Additionally, Biondi et al. (2018) reported that Wolbachia bacterial infection might benefit *T. absoluta* by affecting its reproduction. Spinosad, derived from *Saccharopolyspora spinosa*, has also been effective in controlling *T. absoluta* (Baniameri and Cheraghian, 2012; Caparros Megido et al., 2012). Studies by El-Ghany et al. (2016) and Aynalem et al. (2022) highlighted the significant pathogenicity of entomopathogenic fungi and bacteria, such as *B. bassiana*, *M. anisopliae*, and *B. thuringiensis*, against *T. absoluta* in field conditions.

*B. bassiana* has demonstrated potential as an epiphytic, endophytic, and insecticidal agent in greenhouse environments (Klieber and Reineke, 2016). It can colonize tomato plants endophytically, providing effective control against the tomato leaf miner (Allegrucci et al., 2017). Ibranhim et al. (2017) suggested that *M. anisopliae* and *B. bassiana* conidia are promising for short-term *T. absoluta* control. Further studies by Tadele and Emana (2017) and Ayele et al. (2020) confirmed the high insecticidal activity of these fungi in Ethiopian laboratories and glasshouses.

Entomopathogenic bacteria, such as *B. thuringiensis*, can induce diseases in various insect pests. *B. thuringiensis* (Bt) is a Gram-positive, spore-forming bacterium that produces δ-endotoxin, hemotoxin, and vegetative proteins. Since the 1950s, Bt has been used as a natural insecticide to control specific insect pests. The toxic genes on the Bt plasmid, which encode crystal proteins, are vital for developing pest-resistant genetically modified plants. This makes Bt a significant biopesticide worldwide, with targeted insecticidal activity that minimizes harm to non-target organisms. Researchers have classified numerous crystal protein-coding genes in Bt, grouped based on their sequences. Different Cry genes produce toxins targeting specific insect groups, including lepidopterans, coleopterans, nematodes, and dipterans. Bt strains can carry multiple crystal toxin genes, suggesting a mechanism for gene transfer between strains, enhancing toxin diversity (Aynalem, 2022).

### 4.2 Entomopathogenic nematode

Entomopathogenic nematodes (EPNs) are cosmopolitan, non-segmented, cylindrical, and elongated organisms playing a crucial role in biological control (Hominick et al., 1996). These nematodes are classified into 23 families, with seven families, including Mermithidae, Tetradonematidae, Allantonematidae, Phaenopsitylenchidae, Sphaerulariidae, Heterorhabditidae, and Steinernematidae, containing the most effective species for insect pest control (Lacey and Georgis, 2012). EPNs have shown high efficacy in controlling *T. absoluta* larvae, achieving 79–100% mortality under laboratory conditions (Batalla-Carrera et al., 2010). Leaflet bioassays revealed 77–92% larval nematode infection within the galleries, while pot experiments demonstrated an 87–95% reduction in *T. absoluta* infection (Batalla-Carrera et al., 2010).

Two nematode species, *Heterorhabditis bacteriophora* and *Steinernema carpocapsae*, caused 92–96% and 89–91% larval mortality under laboratory conditions, respectively. These species also achieved 48–51% control of *T. absoluta* in greenhouse conditions (Kamali et al., 2018). Additionally, *H. bacteriophora*, *S. carpocapsae*, and *Steinernema feltiae* showed significant insecticidal activity, with 77–97.4% mortality on *T. absoluta* larvae (Van Damme et al., 2016). EPNs utilize mutualistic intestinal bacteria to eliminate insect pests (Boemare, 2002; De Waal et al., 2011; Van Damme et al., 2016).

Their use in pest management is widespread and effective across various taxa, including similar Lepidopterans like the false codling moth (*Thaumatotibia leucotreta*), codling moth (*Cydia pomonella*), and sugarcane borer (*Eldana saccharina*) (De Waal et al., 2011; Malan et al., 2011; Nthenga et al., 2014). Recent research has confirmed that *S. feltiae*, *S. carpocapsae*, and *H. bacteriophora* are effective against all larval instars of *T. absoluta* (Kamali et al., 2018). These findings indicate that EPNs have significant potential in managing *T. absoluta* and can be integrated into pest management strategies.

Entomopathogenic fungi, such as *B. bassiana* and *M. anisopliae*, are often preferred over entomopathogenic bacteria, viruses, and nematodes for controlling *T. absoluta* due to their broader host range and effective modes of action. These fungi can infect *T. absoluta* through direct contact or ingestion, providing effective control against both larvae and adults. They are environmentally safe, adaptable to various conditions, and less prone to resistance development compared to other entomopathogens (Aynalem, 2022). Furthermore, fungi offer versatility in formulation and application methods, making them suitable for integrated pest management strategies. Entomopathogenic fungi present promising prospects for sustainable and effective *T. absoluta* management.

## 5 Mode of action of entomopathogenic fungi on *Tuta absoluta*

Entomopathogenic fungi are a group of fungi that specifically infect and kill insect pests. These fungi have evolved intricate strategies to invade, proliferate within, and ultimately cause the death of their insect hosts. The mode of action of entomopathogenic fungi involves several key steps:

### 5.1 Attachment and adhesion

**Figure 4:**
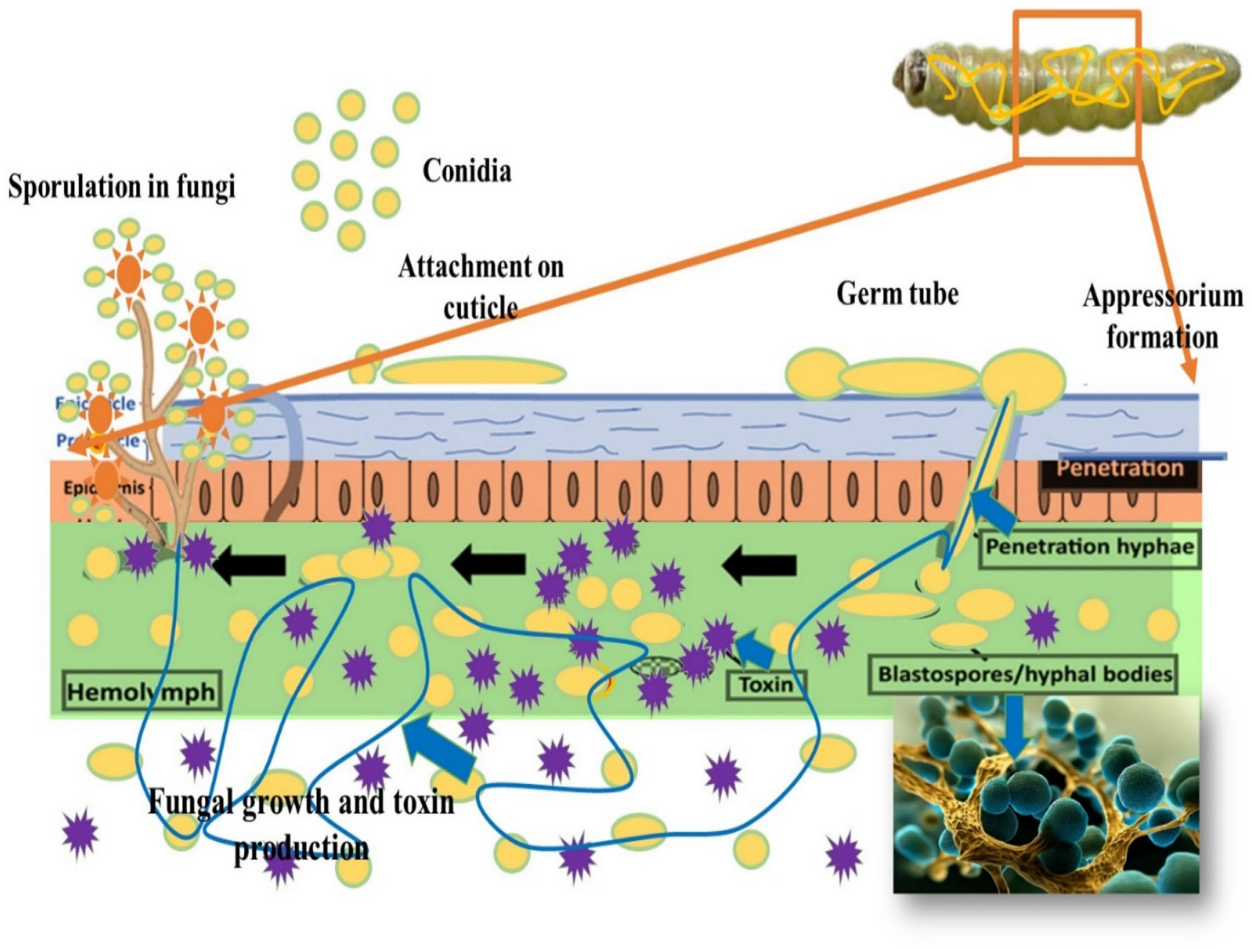
Mode of action of entomopathogenic fungi on *T. absoluta.*

Entomopathogenic fungi possess specialized spores called conidia, which are adapted to attach to the insect’s cuticle. These conidia feature structures such as hydrophobins or other adhesive proteins that facilitate binding to the insect’s exoskeleton (see Figure 4). This attachment is crucial for initiating the infection process and subsequent penetration into the insect’s body. The hydrophobic nature of these structures ensures that the spores adhere firmly to the insect’s surface, even under humid conditions, establishing the fungal infection effectively (Vidhate et al., 2023). This initial adhesion is a critical step in the process through which entomopathogenic fungi infect their insect hosts.

### 5.2 Penetration

Once attached to the insect’s cuticle, the conidia of entomopathogenic fungi undergo germination, developing specialized structures essential for host penetration. One such structure is the appressorium, a highly specialized cell type that exerts mechanical force and enzymatic activity to breach the insect cuticle. Appressoria are pressure-sensitive cells that apply physical pressure to penetrate the insect cuticle. Additionally, they secrete enzymes, including chitinases and proteases, which degrade the cuticle’s components. Chitinases target chitin, a major component of the cuticle, while proteases break down cuticular proteins. This combined mechanical and enzymatic action allows the fungal hyphae to penetrate the insect’s body, overcoming the protective barrier of the cuticle and establishing infection within the host (Ma et al., 2024).

### 5.3 Colonization and proliferation

After penetrating the insect’s cuticle, the entomopathogenic fungus enters the hemocoel, the body cavity containing hemolymph. Inside the hemocoel, the fungus undergoes a transformative growth phase, developing filamentous hyphae. These hyphae extend and spread throughout the hemocoel, invading various tissues and organs of the insect host. As the hyphae proliferate, they disrupt normal physiological functions and cause extensive damage to internal structures. The fungal hyphae absorb nutrients from the insect’s tissues, depriving the host of essential resources necessary for survival. This invasive process highlights the pathogenic nature of entomopathogenic fungi and their ability to efficiently colonize and exploit their insect hosts. Ultimately, the fungal infection progresses, consuming vital host tissues and resources, leading to the death of the insect (Ma et al., 2024).

### 5.4 Nutrient utilization and host tissue degradation

Nutrient utilization and host tissue degradation by entomopathogenic fungi are critical phases in the infection process. Once inside the insect’s body, the invading fungal hyphae secrete various enzymes that facilitate tissue degradation and nutrient acquisition. Proteases and lipases play pivotal roles in this process. Proteases target proteins, cleaving them into smaller peptides and amino acids, which breaks down structural and functional proteins within the host’s body. Lipases hydrolyze lipids, accessing lipid reserves and membrane-bound lipids, which are essential components of cell membranes and storage tissues in insects (Quesada-Moraga et al., 2024).

This enzymatic activity leads to significant degradation of host tissues, disrupting normal physiological functions. Vital organs and structures, such as muscles and fat bodies, are progressively broken down by the fungal hyphae, releasing nutrients required for fungal growth and reproduction. This process exemplifies the parasitic nature of entomopathogenic fungi, as they sustain their growth and propagation by harnessing host-derived nutrients. The disruption of normal physiological functions due to tissue degradation contributes to the progression of the fungal infection and eventual mortality of the insect (Liu et al., 2023).

### 5.5 Immune evasion

Immune evasion is a critical adaptation employed by entomopathogenic fungi to overcome the insect’s immune defense and establish successful infections. These fungi have evolved sophisticated strategies, including the production of secondary metabolites, to evade or suppress the host’s immune response.

One key mechanism involves the secretion of secondary metabolites that have immunomodulatory effects. These metabolites can disrupt the recognition and activation of immune cells, such as haemocytes, which are the main cellular defense against pathogens. Some metabolites directly inhibit immune responses, such as phagocytosis (the engulfment of pathogens by immune cells) or the production of antimicrobial peptides. By impairing these immune mechanisms, the fungi can proliferate and spread within the insect’s body without encountering effective cellular defense (Ma et al., 2024).

Furthermore, entomopathogenic fungi may secrete compounds that disrupt signaling pathways involved in immune activation, dampening the insect’s ability to mount a robust immune response. This ability to evade or suppress the host’s immune defense is critical for the pathogenicity and successful colonization of the insect host. By manipulating the insect’s immune system through the production of specific secondary metabolites, these fungi can establish infections and exploit host resources for growth and reproduction.

### 5.6 Systemic effects and death

As entomopathogenic fungi establish and progress through infection within the insect host, they induce systemic effects that ultimately culminate in the death of the host organism. These effects arise from the relentless growth and metabolic activity of the fungal hyphae within the insect’s body (Mahanta et al., 2023).

The fungal hyphae proliferate and extensively colonize the insect’s tissues, actively consuming and depleting host nutrients, including proteins, carbohydrates, and lipids. This nutrient drain deprives the insect of essential resources necessary for sustaining life functions and physiological processes. The invasive growth of fungal hyphae disrupts the integrity and function of vital organs and tissues within the insect’s body, leading to organ failure and impairing critical physiological processes such as digestion, circulation, and metabolism (De Fine Licht et al., 2024).

Entomopathogenic fungi frequently disrupt the insect’s molting process, which is crucial for growth and development. The presence of fungal hyphae can disrupt the synthesis and release of molting hormones, leading to improper or failed molting cycles. This hampers the insect’s ability to shed its exoskeleton and grow, ultimately compromising its survival (Yang et al., 2023).

During the course of infection, entomopathogenic fungi produce various metabolic by-products and toxins. The accumulation of these toxic metabolites within the insect’s body contributes to physiological stress, cellular damage, and an overall decline in health. After killing the insect host, the fungus produces new spores (conidia) on the cadaver. These spores are released into the environment and can infect new susceptible hosts, completing the fungal life cycle (Lima et al., 2024) (see Figure 4).

## 6 Entomopathogenic fungi: advantages, limitations, and future directions

Entomopathogenic fungi offer several advantages as biocontrol agents for managing insect pests. They are highly specific to insects, exhibiting low toxicity to non-target organisms, including humans and other vertebrates. This specificity makes them suitable for integrated pest management (IPM) strategies, minimizing ecological impact. These fungi are environmentally friendly alternatives to chemical pesticides, as they are naturally occurring organisms that degrade quickly in the environment. They support sustainable pest management approaches that reduce reliance on synthetic chemicals (Sharma et al., 2023; Perumal et al., 2024a).

Entomopathogenic fungi employ multiple modes of action to kill insects, including mechanical penetration, enzymatic degradation, and immune evasion. This multifaceted approach reduces the likelihood of insect resistance development compared to single-mode chemical insecticides (Liu et al., 2023). Some entomopathogenic fungi can persist in the environment for extended periods, providing longer-term pest control benefits. They also demonstrate adaptability to various environmental conditions and host species, enhancing their versatility in pest management programs. Entomopathogenic fungi can be effectively integrated with other pest management tactics, such as cultural practices, biological controls (e.g., predators, parasitoids), and, when necessary, chemical controls. This integration enhances overall pest control efficacy and sustainability (Smagghe et al., 2023).

### 6.1 Challenges of entomopathogenic fungi in pest management

Entomopathogenic fungi, while promising as biocontrol agents, face several challenges that limit their widespread adoption in pest management strategies. Compared to chemical insecticides, entomopathogenic fungi typically exhibit slower action in controlling insect populations. They require time to infect, colonize, and ultimately kill target insects, which may not provide rapid control needed in some agricultural settings (Vivekanandhan et al., 2023).

Environmental sensitivity poses another challenge. Factors such as temperature and humidity significantly influence the efficacy of entomopathogenic fungi. Optimal environmental conditions are crucial for successful fungal infection and proliferation, limiting their effectiveness under adverse conditions (Perumal et al., 2024a). While entomopathogenic fungi are highly specific to insects, their narrow host range can restrict their utility to certain target pests. Some fungi are effective only against specific insect groups or life stages, which limits their broader applicability across diverse pest populations.

The production and formulation of entomopathogenic fungi for commercial use present technical and economic challenges. Large-scale production requires specialized facilities and technologies, making it costly and technically demanding. Improvements in production methods and formulation technologies are necessary to enhance the practicality and cost-effectiveness of using these fungi in pest management (Jaronski, 2023; Quesada-Moraga et al., 2024). Moreover, regulatory approval for entomopathogenic fungi as biopesticides can be complex and time-consuming. The process involves rigorous evaluation of safety and efficacy data, which adds to the challenges of bringing these products to market and integrating them into agricultural practices. Addressing these challenges through research and innovation will be essential to maximize the potential of entomopathogenic fungi in sustainable agriculture and integrated pest management programs.

### 6.2 Advancing entomopathogenic fungi in pest management

Entomopathogenic fungi represent a promising avenue for sustainable pest management, yet advancing their application requires addressing several key areas of research and development (Qin et al., 2023). Efforts should prioritize enhancing formulation technologies to improve the stability, shelf-life, and application methods of entomopathogenic fungi (Bhattacharyya et al., 2023). Innovations in encapsulation, adjuvants, and targeted delivery systems are crucial for maximizing efficacy and practicality in diverse environmental conditions. Expanding the host range and efficacy of entomopathogenic fungi through genetic and ecological studies is essential. Genetic engineering can potentially enhance traits such as virulence and environmental tolerance, broadening the spectrum of pests these fungi can effectively control.

Optimizing the integration of entomopathogenic fungi with other pest management tactics, including biological controls and cultural practices, will enhance overall efficacy and sustainability (Smagghe et al., 2023). Continued research is needed to develop integrated pest management strategies that synergistically combine these approaches. Comprehensive environmental monitoring and impact assessments are critical to ensure the safe and sustainable use of entomopathogenic fungi across different ecosystems. Understanding their persistence and ecological interactions is vital for minimizing unintended environmental consequences.

Streamlining production processes, reducing costs, and navigating regulatory pathways are essential for the successful commercialization and widespread adoption of entomopathogenic fungi in agricultural and urban settings (Lankinen et al., 2024). Overcoming these hurdles will facilitate their integration into mainstream pest control practices (Ahmed et al., 2024). Entomopathogenic fungi offer significant potential as effective, environmentally friendly tools for pest management. Addressing current challenges and exploring these future directions will be instrumental in realizing their full potential and promoting sustainable agriculture worldwide.

## 7 Conclusion and perspectives

Entomopathogenic microorganisms, such as bacteria, fungi, and viruses, present promising prospects for controlling *T. absoluta*, a notorious pest of tomato crops. Extensive studies have underscored the effectiveness of various entomopathogens, including *B. thuringiensis* (Bt), *B. bassiana*, *M. anisopliae*, and nucleopolyhedroviral viruses (NPVs), against both larvae and adults of *T. absoluta*. Utilizing entomopathogens offers several advantages in insect pest management. Entomopathogens are highly specific to insects, exerting minimal impact on non-target organisms, which positions them as environmentally friendly alternatives to chemical pesticides. Moreover, entomopathogens employ diverse modes of action such as direct infection, toxin production, and physiological interference with insect hosts. However, the successful application of entomopathogens for *T. absoluta* control necessitates addressing several challenges. These include optimizing application methods to enhance efficacy under varying environmental conditions, improving formulation stability to prolong shelf-life and efficacy, and comprehensively understanding their interactions with environmental factors.

Future directions in entomopathogens research involve exploring novel strains or combinations of entomopathogens, developing integrated pest management (IPM) strategies that synergize entomopathogens with other pest control methods, and innovating delivery systems to ensure consistent and reliable pest suppression. Entomopathogens hold significant promise as sustainable tools for managing *T. absoluta*, offering effective alternatives to synthetic pesticides while promoting environmentally friendly agricultural practices. Continued research and innovation are imperative to fully harness the potential of entomopathogens within integrated pest management programs aimed at sustainable agriculture.

## Data availability statement

The information supporting this review article is fully contained within the article itself.

## Author contributions

PV: Conceptualization, Data curation, Formal analysis, Funding acquisition, Investigation, Methodology, Project administration, Resources, Software, Supervision, Validation, Visualization, Writing – original draft, Writing – review & editing. KS: Data curation, Formal analysis, Methodology, Software, Validation, Visualization, Writing – original draft, Writing – review & editing. PS: Software, Validation, Writing – original draft, Writing – review & editing. KP: Conceptualization, Funding acquisition, Supervision, Validation, Writing – original draft, Writing – review & editing.
